# Using media to impact health policy-making: an integrative systematic review

**DOI:** 10.1186/s13012-017-0581-0

**Published:** 2017-04-18

**Authors:** Lama Bou-Karroum, Fadi El-Jardali, Nour Hemadi, Yasmine Faraj, Utkarsh Ojha, Maher Shahrour, Andrea Darzi, Maha Ali, Carine Doumit, Etienne V. Langlois, Jad Melki, Gladys Honein AbouHaidar, Elie A. Akl

**Affiliations:** 1 Center for Systematic Review for Health Policy and Systems Research, American University of Beirut, Beirut, Lebanon; 2 Department of Health Management and Policy, Faculty of Health Sciences, American University of Beirut, Beirut, Lebanon; 3 Department of Health Research Methods, Evidence, and Impact (HE&I), McMaster University, Hamilton, Canada; 4 Imperial College London, London, UK; 5 Al-Makassed Hospital, Jerusalem, Palestine; 6 AUB GRADE Center, Clinical Research Institute, American University of Beirut, Beirut, Lebanon; 7 Department of Nutrition and Food Sciences, Faculty of Agricultural and Food Sciences, American University of Beirut, Beirut, Lebanon; 8 Leeds Institute of Health Sciences, Faculty of Medicine and Health, University of Leeds, Leeds, UK; 9 Department of Health Promotion and Community Health, Faculty of Health Sciences, American University of Beirut, Beirut, Lebanon; 10 Alliance for Health Policy and Systems Research, World Health Organization, Geneva, Switzerland; 11 Department of Communication Arts, Lebanese American University, Beirut, Lebanon; 12 Hariri School of Nursing, American University of Beirut, Beirut, Lebanon; 13 Department of Internal Medicine, American University of Beirut, P.O. Box 11–0236, Riad-El-Solh Beirut, 1107 2020 Beirut Lebanon

**Keywords:** Media interventions, Media campaigns, Health communication, Health policy-making, Systematic review

## Abstract

**Introduction:**

Media interventions can potentially play a major role in influencing health policies. This integrative systematic review aimed to assess the effects of planned media interventions—including social media—on the health policy-making process.

**Methods:**

Eligible study designs included randomized and non-randomized designs, economic studies, process evaluation studies, stakeholder analyses, qualitative methods, and case studies. We electronically searched Medline, EMBASE, Communication and Mass Media Complete, Cochrane Central Register of Controlled Trials, and the WHO Global Health Library. We followed standard systematic review methodology for study selection, data abstraction, and risk of bias assessment.

**Results:**

Twenty-one studies met our eligibility criteria: 10 evaluation studies using either quantitative (*n* = 7) or qualitative (*n* = 3) designs and 11 case studies. None of the evaluation studies were on social media. The findings of the evaluation studies suggest that media interventions may have a positive impact when used as accountability tools leading to prioritizing and initiating policy discussions, as tools to increase policymakers’ awareness, as tools to influence policy formulation, as awareness tools leading to policy adoption, and as awareness tools to improve compliance with laws and regulations. In one study, media-generated attention had a negative effect on policy advocacy as it mobilized opponents who defeated the passage of the bills that the media intervention advocated for. We judged the confidence in the available evidence as limited due to the risk of bias in the included studies and the indirectness of the evidence.

**Conclusion:**

There is currently a lack of reliable evidence to guide decisions on the use of media interventions to influence health policy-making. Additional and better-designed, conducted, and reported primary research is needed to better understand the effects of media interventions, particularly social media, on health policy-making processes, and the circumstances under which media interventions are successful.

**Trial registration:**

PROSPERO 2015:CRD42015020243

**Electronic supplementary material:**

The online version of this article (doi:10.1186/s13012-017-0581-0) contains supplementary material, which is available to authorized users.

## Background

Media interventions are defined as organized and purposive activities that utilize a variety of media channels to inform, persuade, or motivate populations [1]. In health care, media interventions can convey health-related information including research evidence to the public, policymakers, and health professionals [2–6]. They can also influence individual health behaviors [7]. For instance, media campaigns were shown to be effective in decreasing tobacco uptake, reducing alcohol-impaired driving and alcohol-related crashes and influencing health services utilization [8–10].

In the area of policy-making, media can contribute to setting the agenda for the press, the public, and policymakers through highlighting what issues are newsworthy at a particular time [11]. Media can also influence how the public and policymakers view or think about certain issues through selecting some aspects of a perceived reality and making them more salient in a communicating text [12]. Another way media can influence policymakers is through shaping public opinion, which in turn, exerts pressure on policymakers to respond [13]. For instance, media advocacy is known as a popular strategy in public health that can assist in increasing public awareness and mobilizing decision-makers for policy change [14, 15].

When examining the various functions that media interventions can play in the health field, it is important to consider new interactive information and communication platforms, particularly social media including blogs, social networking sites, and interactive websites. While traditional media, mainly television, still account for a large audience, the influence of social media is constantly increasing and cannot be ignored [16, 17]. Social media increase user interaction, provide peer support, and extend access to health interventions [16, 17]. Social media also bring a new dimension to health care as they provide the public, patients, and health professionals with a platform to exchange on different health matters potentially affecting population health outcomes [18].

A number of systematic reviews on the impact of media interventions on health behavior and their use in increasing awareness and education exist [16, 19, 20]. However, to our knowledge, there is no systematic review assessing the role of media interventions in the different stages of health policy-making. Our objective is to better inform those considering the use of media interventions to influence health policy-making. Therefore, we conducted this integrative review to assess the effects of planned media interventions on the health policy-making process.

## Method

### Protocol and registration

A protocol for this review is registered in PROSPERO International prospective register of systematic reviews under registration number PROSPERO 2015:CRD42015020243 [21]. While we had planned in the protocol to start the search with the year 2000, following our discussion with a media expert, we changed the date to the year 2005—the year of the rise of “web 2.0”. Web 2.0 allowed users of the web to interact and generate content especially through social media [22]. Around that time, traditional mainstream media also started to more seriously integrate social media within their operations.

### Methodology

We used the integrative review methodology that aims to include a range of studies from different methodological approaches (both experimental and non-experimental). We followed the five stages of an integrative review by Whittmore et al.: problem formulation, literature search, data evaluation, data analysis, and data presentation [23]. This methodology is meant to protect against bias, enhance rigor, and improve accuracy of conclusions [23].

### Problem formulation

Herein the definitions of terms used in the research question:

#### Media interventions

Media interventions are described as organized and purposive activities that utilize a variety of media channels to inform, persuade, or motivate populations [1]. Given that the goal of this review was to inform those interested in using media interventions to affect health policy-making, we restricted our eligibility to studies where the primary purpose of using media was to affect policy-making (i.e., media as planned intervention). For example, we excluded studies where unplanned media coverage influenced health policy-making or where media coverage followed a health policy change. To make this distinction, we adopted the terminology used by a published Cochrane review on a related topic—effects of mass media on the utilization of health services—to distinguish between the two approaches: planned campaigns/interventions and unplanned media coverage [10].

#### Public policy

Public policy referred to government policy such as any statement or position taken by the government or government departments [13]. We only considered public policies pertaining to health. We adopted the stages heuristic framework that divides the public policy process into five stages: agenda-setting, policy formulation, adoption, implementation, and evaluation. Agenda-setting is the stage during which issues or subjects reach the policy agenda and get the attention of policymakers. In the formulation and adoption stages, legislatures and other decision-makers design policies and adopt policy solutions in the form of legislation or rules. In the implementation stage, governments carry out an adopted policy and resources are mobilized. Finally, the evaluation stage aims at assessing whether policies have achieved their intended objectives [24, 25].

#### Outcomes of interest

Our outcomes of interest were the impact of the intervention on the different stages of the policy process, as defined above: agenda-setting, policy formulation, adoption, implementation, and evaluation. We only included studies that assessed the effects of media on policy outcome. For this purpose, we used a framework that provides indicators to assess the influence of media advocacy on policy outcomes. Some of the indicators include speeches and statements, mentions in official documents, new policy or legislation, and increased enforcement of a policy. We did not consider surrogate outcomes such as the impact on media coverage, community, or public opinion [26].

### Literature search

#### Eligibility criteria

We considered as eligible for this integrative review the following:

- Study designs: randomized studies, non-randomized studies, economic studies, process evaluation studies, stakeholder analyses, qualitative studies, and case studies. We excluded editorials, commentaries, news articles, letters, conference papers, proposals, reviews, and studies published only in abstract format;
- Planned media interventions (e.g., advocacy activities, media campaigns) implemented as stand-alone or as part of multicomponent interventions, including social media (Facebook, Twitter, blogs), broadcast media (radio, television), print media (newspapers, newsletters, magazines, leaflets, posters, and pamphlets), and electronic media (websites). The media interventions should have targeted a specific population such as specific communities, policymakers, groups, or associations;
- Setting: any country, state, or community;
- Studies that assessed the impact of media interventions on one of the policy stages as defined in the stages heuristic framework: agenda-setting (setting the policy agenda and establishing priorities), policy formulation, adoption, implementation, and evaluation [24]. We excluded studies that did not formally assess the association between media exposure and policy-making (e.g., assessed surrogate outcomes such as increasing exposure, engagement, and preferences of the public for certain policies).

#### Search strategy

We searched Medline, EMBASE, Communication and Mass Media Complete, Cochrane Central Register of Controlled Trials (CENTRAL), and the WHO Global Health Library. We developed the search strategies used in the different databases in consultation with an expert librarian. We used both free text search terms and MeSH terms (see details for the different electronic databases in Additional file 1). We ran the search from January 2005 until June 2015. We did not restrict the search to specific languages. We also screened the reference lists of included studies to retrieve additional studies, and we contacted experts in the field (including authors of included studies) to get additional material.

#### Selection process

We imported the search results into Endnote X7 and removed duplicates. Before starting the selection process, and in order to ensure its reliability, all the reviewers participated in a calibration exercise using a randomly chosen sample of 100 citations.

The selection process consisted of two stages:

1. Title and abstract screening: Teams of two reviewers (LBK, MS, AD, MA, CD) used the eligibility criteria to screen titles and abstracts of identified citations in duplicate and independently for potential eligibility. Then, they retrieved the full-texts for citations judged as potentially eligible by at least one of the two reviewers.
2. Full-text screening: Teams of two reviewers (LBK, NH, YF, UO, MS) used the same eligibility criteria to screen the full-texts in duplicate and independently for eligibility. At this stage, the two reviewers compared results and resolved disagreement by discussion. The reviewers aimed not to force consensus, and when consensus could not be reached, a third reviewer (FJ or EAA) made the final decision. We used standardized and pilot-tested screening forms. We documented the reason for study exclusion.

We used Fleiss’ Kappa coefficient to calculate agreement between reviewers for full-text screening. We used the following values to judge the degree of agreement: 0.21–0.40 for fair agreement, 0.41–0.60 for moderate agreement, 0.61–0.80 for substantial agreement, and 0.81–1.00 for almost perfect agreement [27].

#### Data evaluation

Throughout the process of data abstraction, two reviewers abstracted data from eligible studies in duplicate and independently. We used standardized and piloted data abstraction forms. We conducted a calibration exercise on a randomly chosen sample to ensure adequate agreement. The reviewers resolved their disagreements by discussion or with the help of a third reviewer when consensus could not be reached.

We collected the following information from each included evaluation study: objectives, type of study design, details of the methodology, study setting (health topic and jurisdiction), population, characteristics of the media intervention, type of media used, outcome assessed, study theme, results, and limitations of the study. We abstracted from each included case study the study’s name and country, health topic, type of media used, characteristics of the media intervention, and policy outcome.

As part of data evaluation, we appraised the methodological quality of included studies using tools appropriate to the study design. Two reviewers (LBK, NH) assessed the quality of included studies independently and resolved disagreements through discussion or with the help of a third reviewer when needed. We considered the following tools for assessing the risk of bias (quantitative) and quality of reporting (qualitative) of the included studies:

- The Cochrane Risk of Bias tool for randomized trials;
- A modified version of the Cochrane Risk of Bias tool for non-randomized studies (A Cochrane Risk Of Bias Assessment Tool: for Non-Randomized Studies of Interventions (ACROBAT-NRSI);
- The Cochrane Effective Practice and Organization of Care (EPOC) risk of bias criteria for controlled before and after studies and interrupted time series;
- The Critical Appraisal Skills Program (CASP) tool for qualitative studies;
- A tool adapted from Lotfi et al. [28] to assess the methodological features of quantitative studies using surveys including the following criteria: sample size calculation, reporting of a sampling frame, the sampling method, the response rate, and the validity of the survey.

### Data analysis and presentation

We stratified the studies based on the stages of the policy-making process. We conducted thematic analysis of all papers reflecting the role of media as reported in each study and presented the results in a narrative summary by stage. Two reviewers (LBK, NH) identified the themes. A third reviewer (GH) settled any discrepancy. We did not identify a mix of quantitative and qualitative data for any of the stages we examined; therefore, we could not conclude whether the included studies confirmed, refuted, or complemented each other [29].

As for case studies, we summarized them in a table (see Additional file 2) listing the study’s name and country, health topic, type of media used, and characteristics of the media intervention and policy outcome.

## Results

### Study selection

**Fig. 1:**
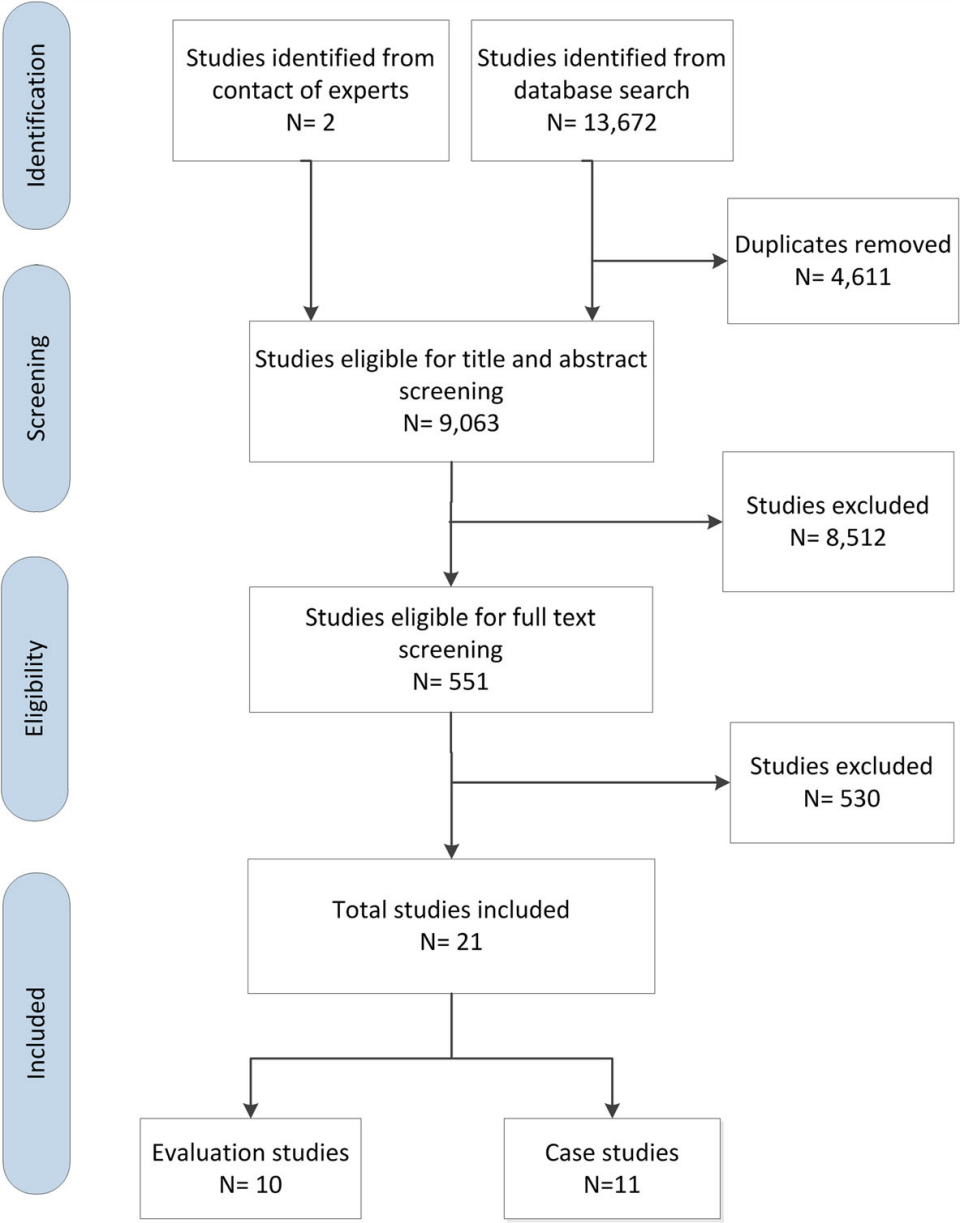
PRIMSA flowchart for study selection process

Figure 1 shows the PRISMA flow chart summarizing the study selection process. Out of 13,674 citations, we identified 21 eligible studies: 10 evaluation studies and 11 case studies. We excluded 530 articles at the full-text screening phase for the following reasons: not an intervention of interest (*n* = 148), not about public health policy (*n* = 155), not an outcome of interest (*n* = 164), and not the design of interest (*n* = 63). Additional file 2 provides detailed descriptions of the 11 case studies [30–40]. We provide below the characteristics and results of the 10 evaluation studies [41–50].

### Characteristics of the included evaluation studies

Table 1 describes the characteristics of the 10 eligible evaluation studies [41–50], including study design, setting, population, media intervention (type and nature), and outcome assessed.

**Table 1:** Characteristics of included studies

Haq 2010 [41]	
Study setting	Health topic: maternal and newborn health Pakistan
Population	Target of media intervention: district-level health officials/policymakers
Characteristics of the media intervention	TV talk show: The aim of the TV talk show was to get on-camera commitments from three keys belonging to district health policy. The show started with a viewing of the documentary after which the host invited discussion by the participants. The discussion started with the panelists providing information on their plans to improve the MNH situation in their area. The host also invited members of the audience to raise their questions and concerns publicly on the show. Date: The program series was aired from April to June 2007. Level: national Type of media: broadcast media (television) Organizer: Pakistan Initiative for Mothers and Newborns (PAIMAN); a project designed to improve MNH in 10 districts.
Study design	Qualitative method Data sources: interviews with 20 out of 31 participants
Outcome	Change in policy behavior/approach
Vasudevan 2009 [48]	
Study setting	Health topic: road safety State of NE, US
Population	Target of media intervention: the public
Characteristics of the media intervention	Media and enforcement campaign: The media and enforcement campaign is part of “Click it or ticket program”. The media campaign consisted of -Paid media (paid television and radio advertisements) -Earned media (ride-along with law enforcement officers, coverage of press event kick offs and upcoming enforcement events, newspaper coverage of enforcement campaign) Date: May 2003, May 2004, and May 2005 Level: state Type of media: broadcast media (television and radio), print media (newspapers) Organizer: the state
Study design	Quantitative method: before and after design Data sources: -Seat belt usage observations conducted at 50 sites in Nevada
Outcome	Seat belt usage rates
Rehnman 2005 [47]	
Study setting	Health topic: alcohol control Norrmalm (inner-city area), Stockholm, Sweden
Population	Target of media intervention: the public
Characteristics of the media intervention	Beer campaign: Media intervention was part of a multi-component intervention named “The beer campaign” that consisted of a series of activities involving information/training, media advocacy, and monitoring. In 1998, The intervention also included meetings with merchants, sending post card to parents, training of staff, and media advocacy. In 1999, a follow-up study indicated no improvements, which was followed by a renewed intervention, with added components: merchants received individual feedback on sales in their shops, and they committed themselves to buy back the beer that was sold to the students in the study. The media advocacy consisted of presenting the baseline purchase study and follow-up study a year later at press conferences. The results were presented in several newspapers, local radio, and television. Contacts were maintained with both local and national media during the following year. Date: 2 years (1998–2000) Level: county Type of media: broadcast media (television and radio), print media (newspapers) Organizer: The STAD Project (Stockholm Prevents Alcohol and Drug Problems)
Study design	Quantitative method: controlled before and after design Data sources: -Purchase studies (controlled before and after design)
Outcome	Successful beer purchase attempt
Sivaneswaran 2011 [46]	
Study setting	Health topic: water fluoridation/dental hygiene New South Wales towns, Australia
Population	Target of media intervention: the public
Characteristics of the media intervention	Community education program: Community education program including the organization of a public forum and the use of media. The use of media involved Fluoridation information kits prepared by the NSW Health Department containing relevant information on water fluoridation were also provided to the local media, Council offices as well as to those present at the public forums. In addition, a significant amount of information on water fluoridation was also provided to the community via the local newspapers, radio and television. Early during the campaign, NSW Health Department representatives met with the editor of the Mudgee Guardian to ensure balanced and impartial reporting of articles or editorial letters relating to water fluoridation and the editor was also invited to the forum. Date: February 2005 Level: province Type of media: broadcast media (television and radio), print media (newspapers) Organizer: state (Mid-Western Regional Council, NSW Health Department)
Study design	Quantitative design Data sources: -Surveys completed from telephone interviews -Surveys completed from face-to-face interviews
Outcome	- Support of the water fluoridation measure and adoption of fluoridation policy
Leurer 2013 [50]	
Study setting	Health topic: nursing education Saskatchewan, Canada
Population	Target of media intervention: Government/policymakers at the government level and the public
Characteristics of the media intervention	Media advocacy: Nursing stakeholders quickly reacted to the “January 21st” policy announcement by using media advocacy to convey messages designed to exert pressure on the government to reconsider the new policy. Media advocacy including -Framing and releasing of news releases, letters to the editor, press releases -Wide press coverage of nursing students protests -Media interviews Date: 3-month period following January 21, 2000 Level: province Type of media: print media Organizer: nursing stakeholders
Study design	Qualitative media analysis: Data sources: - The print media sources included articles, editorials, and letters to the editor from The Leader-Post, the daily newspaper in Saskatchewan’s provincial capital Regina.
Outcome	- Change in government policy
Harwood 2005 [44]	
Study setting	Health topic: Alcohol control State of LA, USA
Population	Target of media intervention: policymakers and the general public
Characteristics of the media intervention	Media advocacy was an integral component of the coalitions’ work as they sought to affect public and legislative awareness and opinions about underage drinking issues. Some examples include solicited media coverage of rallies at the state capital, community information meetings, youth-lead community activities, and media interviews with coalition members for feature stories on underage drinking. Date: 1997–2004 Level: state Type of media: print media (newspapers) Organizer: coalitions by the Robert Wood Johnson Foundation to reduce underage drinking
Study design	Quantitative design Data sources: - Media data - Legislative data
Outcome	- Passage of alcohol bills
Gardner 2010 [42]	
Study setting	Health topic: Access to health care State of CA, USA
Population	Target of media intervention: policymakers and the general public
Characteristics of the media intervention	Media advocacy activities including a combination of strategies: -launching websites -creating videos (some videos described the role of primary care clinics in specific regions of the state that were distributed to television stations and other venues) -developing articles and letters to the editors for local and statewide newspapers -working with local radio and television outlets -television and radio interviews -developing member clinic capacity to conduct media outreach Date: the fund started in 2001 for 3 years and was renewed in 2004 and 2007 Level: state Type of media: print media (newspaper, brochures, newsletters), broadcast media (television, radio, video), electronic media (websites) Organizer: Clinics Consortia
Study design	Quantitative and qualitative method Data sources: -Annual Policy Advocacy Activities Worksheet -Annual Grantee Interviews -Policy Maker and Community Leader Awareness Survey -Media Representative and Consultant Interviews
Outcome	Effectiveness of media advocacy activities (increasing policymakers awareness, achieving a policy change or increased funding to clinics)
Gowda 2008 [43]	
Study setting	Health topic: water fluoridation Northland, New Zealand
Population	Target of media intervention: the general public and policymakers
Characteristics of the media intervention	Fluoridation advocacy program including policy advocacy, community action projects and media advocacy. Media advocacy included proactive and reactive media releases in local newspapers, letters to the editors of various communities and local newspapers. Other channels used were the provision of information on the District Health Boards (DHB) websites, participation in newspapers and radio interviews (e.g., talkback shows on the radio) and information through school newsletters. Positive messages were released to the media from Northland DHB and a network of supporters was established to respond to any letters or other initiatives from opponents of community water fluoridation. The timing of the positive publicity and reopening of the fluoridation issue was important in gaining support from other health professionals, Māori health providers, PHOs and the community. Date: 2005 Level: district Type of media: print media (newspapers), broadcast media (radio), electronic media (District Health Boards websites) Organizer: Northland District Health Board
Study design	Quantitative and qualitative methods (process evaluation) Data sources: -Written documents -Field notes from direct observation and participation - -Outcomes of community surveys
Outcome	- Support of the water fluoridation measure and adoption of fluoridation policy
Niederdeppe 2007 [45]	
Study setting	Health topic: Tobacco control State of FL, USA
Population	Target of media intervention: policymakers and the general public
Characteristics of the media intervention	The Florida Tobacco Control Program’s (FTCP) media advocacy strategy, a secondary program component, involved sending press releases and working with reporters to promote FTCP programs, media training for local SWAT leaders and promoting media events coordinated with local SWAT activities. Date: FTCP was launched in 1998 Level: state Type of media: print media and Broadcast media Organizer: Florida Tobacco Control Program (FTCP)
Study design	Quantitative design (event history analysis) Data sources: -Content analysis of news coverage -Surveys
Outcome	- Passage of tobacco product placement ordinances
Rock 2011 [49]	
Study setting	Health topic: food insecurity Canada
Population	Target of media intervention: policymakers and the general public
Characteristics of the media intervention	Media advocacy: The media intervention conveyed the message that food insecurity is a serious population health problem. The media intervention was implemented in an effort to increase public awareness about the negative effects of poverty on health in conjunction with the publication of a study comparing the perspectives of food-insecure and food-secure Canadians. The intervention included -News conference -Communication with journalists -Video news release (VNR) and DVDs -Media release Date: 2008 Level: national Type of media: print media, broadcast media and electronic media Organizer: University of Calgary
Study design	Qualitative design (content analysis) Data sources: -Media stories (to track media coverage) -Emails (to track reactions from government representatives and social service providers) -Hansard (the traditional name of the transcripts of Parliamentary Debates) -Telephone and face-to-face communication
Outcome	- Reactions of policymakers to media coverage

#### Study design

The designs of the 10 evaluation studies were

- Quantitative (*n* = 7): before and after design (*n* = 2) [47, 48], event history analysis (*n* = 1) [45], media and document analysis (*n* = 1) [44], and cross-sectional survey (*n* = 3) [42, 43, 46]. Two of those studies included qualitative components that were not relevant to our question [42, 43].
- Qualitative (*n* = 3): interviews (*n* = 1) [41], media analysis (*n* = 1) [50], and content analysis (*n* = 1) [49].

#### Setting and population

Except for one study that was conducted in Pakistan [41], the included studies were conducted in high-income countries: USA (*n* = 4), New Zealand (*n* = 1), Australia (*n* = 1), Canada (*n* = 2), and Sweden (*n* = 1). One study targeted policymakers only [41], three targeted the public only [46–48], and six targeted both [42–45, 49, 50].

The health topics were water fluoridation (*n* = 2), alcohol control (*n* = 2), tobacco control (*n* = 1), road safety (*n* = 1), maternal and newborn health (*n* = 1), nursing education (*n* = 1), access to health care (*n* = 1), and food insecurity (*n* = 1).

#### Type of media assessed

All campaigns relied on the use of traditional media (including broadcast and print media); none included social media. Media activities included a combination of paid media and earned media, where the topic received recognition and gained publicity for free. Examples of encountered media activities were advertisements (*n* = 1) [48], news coverage of events and campaigns (*n* = 3) [44, 48, 50], media interviews (*n* = 3) [42, 44, 50], TV talk shows (*n* = 1) [41], creating videos (*n* = 2) [42, 49], launching of websites (*n* = 1) [42], framing and releasing of news and press releases (*n* = 3) [43, 45, 50], letters to the editor (*n* = 3) [42, 43, 46], provision of information kits to media (*n* = 2) [43, 46], press conferences (*n* = 2) [47, 49], and media events (*n* = 1) [45].

#### Nature of the intervention

- In four studies, the media intervention was part of a multi-component intervention, where authors assessed the effect of the whole intervention on policy outcome. Other components included enforcement campaign [48], parent meetings, merchant meetings, visits to shops, postcards to parents, letters to merchants, training of staff [47], policy advocacy and community action projects [43], and a public forum [46].
- In three studies, the media activities were part of a broad intervention program but authors assessed the effect of the media component on policy outcome [42, 44, 45].
- In three studies, the media intervention was implemented alone and the effect of the media on policy outcome was explored [41, 49, 50].

#### Outcomes assessed

- Impact on agenda-setting assessed as change in policy priorities/approach of policymakers and as stimulating inquiries and discussions from policymakers [41, 49].
- Impact on policy formulation assessed as change in content of government policy [50].
- Impact on policy adoption assessed as passage of bills and ordinances [44, 45], as change in policies or increased funding to clinics [42], and as adoption of a new policy [43, 46].
- Impact on policy implementation assessed as increased enforcement of law, in particular change in seat belt usage rates [48] and change in availability of beer to the under-aged [47].

### Methodological appraisal

Additional file 3 summarizes the detailed assessment of the methodological appraisal of the 10 evaluation studies. Using the CASP tool, we judged the qualitative study to be of high quality as it reported on 8 of 10 criteria of the tool [41]. Using the tool adapted from Lotfi et al. [28], we judged the three quantitative survey studies to be at high risk of bias since they suffered from a number of methodological limitations [42, 43, 46]. The same applied to the controlled before and after study [47], using the EPOC risk of bias criteria. We were not able to find tools to assess the methodological quality of the other five studies. So we assessed their limitations narratively (see Additional file 3). Overall, they all suffered from at least one major limitation (mainly related to confounding), so we judged them to be at high risk of bias.

### Effects of media interventions on policy outcomes

Additional file 4 provides themes and results of individual studies. We present below the findings organized according to the stages heuristic framework of the public policy process and present the overarching effect of media on each stage highlighted in italic font [24].

#### Agenda-setting

Two qualitative studies have assessed media interventions as stand-alone interventions and found positive perceptions regarding the impact of media on agenda-setting.

Haq et al. [41] used a qualitative study design to examine the use of *media as accountability tools leading to prioritizing and initiating policy discussions*. The study evaluated a television talk show in Pakistan aiming to get on-camera commitments from key officials and policymakers belonging to district health policy. The interviews with the officials and policymakers 6 months following the TV talk show suggested that this media accountability tool was an “effective” strategy in setting maternal and newborn health as a priority health issue in the targeted districts, as perceived by the interviewees. The effectiveness of television talk show was based on the perceptions of the interviewees [41].

Rock et al. [49] used a content analysis design to qualitatively examine *media as tools to increase policymakers’ awareness* in Canada in 2008. The media intervention intended to convey the message that income-related food insecurity is a serious population health problem. The authors of this study reported that the media intervention generated interest within the Government of Canada and the Government of Alberta, in particular, stimulated discussion in the Alberta Legislature and sparked inquiries from the Senate Committee on Social Affairs Subcommittee on Cities and from a political staff member [49].

#### Policy formulation

Leurer [50] used a qualitative media analysis to examine the role of *media as advocacy tools to influence policy formulation*. The authors of the study reported that media advocacy efforts of nursing stakeholders in Saskatchewan, Canada, increased public pressure that in return led the government to revise its policy that originally intended to impose a 3-year diploma entry requirement despite opposition from the licensing body. They also noted that what happened in Saskatchewan “stands in contrast to the neighboring province of Manitoba where the government’s announcement of a reintroduction of the diploma entry requirement in 2000, within weeks of the Saskatchewan announcement, was implemented” [50].

#### Policy adoption

All the five studies that examined the effect of media on policy adoption were quantitative [42–46]. All five focused on *media as awareness tools leading to policy adoption*. The differences were in the targets of the planned media. All but one study, Harwood et al. [44], suggested that media interventions were successful in achieving the intended goals.

Harwood et al. [44] assessed the association between media attention to alcohol issues and the legislative success of related bills. A detailed analysis of media covers four alcohol policies: bans on minors in bars, increases in alcohol taxes, beer keg registration, and zero tolerance for teen driving under the influence of alcohol. The analysis showed that out of the five bills that successfully passed, four received little or no media attention while all the bills that were defeated received intense media coverage. The authors speculated that press attention may have hindered the passage of bills through mobilizing opponents but they provided no details on those “opponents” or whether they run any counter campaigns. The authors also concluded that press inattention may give stakeholders the opportunity to compromise during negotiations on bill content and wording [44].

Niederdeppe et al. [45] suggested that media awareness efforts implemented as part of the Florida Tobacco Control Program (FTCP) in FL, USA, were effective in generating news coverage and promoting policy change—the passage of the tobacco product placement ordinances (TPPO). TPPO are designed to reduce youth smoking by removing the visual and physical availability of cigarettes, requiring retailers to place cigarettes and other tobacco products behind the counter. The study found that a one-unit increase in news coverage on Students Working Against Tobacco, a part of the FTCP, was associated with a 94% increase in the odds of counties enacting a TPPO (*p* value <0.05). The effect persisted when the analysis controlled for community mobilization and pro-tobacco marketing influences [45].

Two studies (Sivaneswaran et al. and Gowda et al.) assessed the role of *media as awareness tools to gain public support leading to policy adoption* [43, 46]. Sivaneswaran et al. [46] explored the role of a multicomponent education campaign including the use of media to educate and increase awareness about the importance of water fluoridation. The purpose of the campaign was to gain public support before the adoption and implementation of the water fluoridation policy in two rural towns in New South Wales, Australia, in 2005. Thus, the Mid-Western Regional Council commissioned an independent rural research organization to survey households in those two communities to assess their support of the fluoridation. The survey found that newspapers followed by radio and television (*n* = 80) were the most common sources of information (86%). Among those who were informed, 59% indicated that they were supportive of the policy compared with 47% among those not informed. Although the authors do not explicitly provide evidence for the link between public opinion and policy adoption, they make a clear statement supporting that link: “the pro-fluoridation decision was influenced by the majority of community support for the measure” [46].

Gowda et al. [43] evaluated a fluoridation advocacy program implemented in Northland, New Zealand. Media were employed as part of the advocacy program to raise awareness and to promote water fluoridation among the community and decision-makers. The authors reported on a community consultation survey that showed that the public was in favor of fluoridation in two districts of Northland (53 and 56%). Although Gowda et al. did not explicitly provide evidence for the link between public opinion and policy adoption, they clearly stated that obtaining “a simple majority in favor of fluoridation led directly to the District Council’s resolution to fluoridate”*.* The authors of the study judged that positive publicity and messages, the timing, and the reopening of the fluoridation issue were important in gaining support for fluoridation from other health providers, health organizations, and the community [43].

Gardner et al. [42] explored the role of media advocacy as tools for increasing awareness of policymakers on community clinics’ issues and leading to policy adoption. A survey of policymakers, community leaders, and stakeholders in 2003 and 2004 reported that 60% of the 2003 and 42% of the 2004 respondents perceived Clinic Consortia media activities to be “very effective” at increasing their awareness of community clinics’ issues. Nearly all (95%) clinic organizations and associations surveyed rated media as “effective” in increasing policymaker awareness. However, only 20% of the clinic organizations and associations reported that the media were successful in achieving a policy change or increasing funding to the clinics [42]. The effectiveness of media advocacy in this paper was based on the perceptions of the respondents.

#### Policy implementation

Two before and after studies examined *media as awareness tools to improve compliance with laws and regulations.* While the two studies found positive effects on compliance with laws and regulations, media interventions were part of multi-component interventions [47, 48].

Rehnman et al. [47] explored the effects of “the beer campaign” conducted in 1999 and 2000 in Stockholm, Sweden. The campaign aimed to reduce the sale of beer to under-aged youths. The media advocacy campaign was not clearly described and was only one of many components of the intervention that also involved parent meetings, merchant meetings, visits to shops (with feedback and commitment for the 2000 intervention), postcards to parents, letters to merchants, and training of staff. The authors of this study reported on a comparison area but did not specify whether any intervention was implemented there. The study found that in the intervention area, the percentage of successful beer purchase attempts varied from 66% at baseline to 73% at the end of the first year and to 44% at the end of the second year. The values in the comparison area were respectively 60, 86, and 44%. This study provides insufficient evidence on the role of media advocacy in the observed results [47].

Vasudevan et al. [48] assessed the effects of media and a “seat belt” law enforcement campaign in the state of NV, USA, on increasing the seat belt usage rates. The campaign, Click it or Ticket, was conducted during the month of May over 3 years (2003–2005). The authors collected seat belt usage rates among both drivers and passengers pre-campaign (March and April) and post campaign (June and August). They found that the overall seat belt usage rate significantly increased from 73.9 to 78.9% in 2003 (*p* value <0.001), from 81.8 to 86.6% in 2004 (*p* value <0.001), and from 88.4 to 94.8% in 2005 (*p* value <0.001). The authors of this study concluded that “effectively coupling media and enforcement campaigns” led to a “significant increase in seat belt usage” [48].

#### Policy evaluation

No studies were found to examine the role of media in policy evaluation.

## Discussion

### Summary of findings

This integrative systematic review identified 10 eligible studies that evaluated the effects of planned media interventions on the different stages of the health policy-making process, except for the policy evaluation stage. None of the evaluation studies assessed social media interventions. We judged all 10 studies to be at high risk of bias. The findings of the evaluation studies suggest that media interventions may have a positive impact when used as accountability tools leading to prioritizing and initiating policy discussions, as tools to increase policymakers’ awareness, as tools to influence policy formulation, as awareness tools leading to policy adoption (and to gain public support leading to policy adoption), and as awareness tools to improve compliance with laws and regulations. In one study, media-created attention had a negative effect on policy advocacy as it mobilized opponents who defeated the passage of the bills that the media intervention advocated for [44]. One could speculate that the reason for such defeat may be due to more effective counter-media interventions funded and supported by powerful special interests and stakeholders.

In addition, the available evidence suggested a number of factors as possible predictors of the success of the interventions. These include the timing of media publicity, building relationships with the media, the mobilization of opponents, and the concomitant use of other strategies such as enforcement campaigns and community mobilization and engagement [42–44, 46, 49]. This suggests that media interventions do not occur in isolation and a number of factors should be taken in consideration while designing a media campaign for policy change.

### Research gaps in the field

A methodological review of published evidence maps found that the most popular domains used to classify evidence gaps were study design, interventions, setting, population, and outcomes [51]. We present in Table 2 the research gaps relevant to the “use of media to impact health policy-making” according to those domains. Similarly, we used a framework developed by Robinson et al. to discuss the reasons behind the research gaps [52]. Using that framework, we identified three reasons related to the topic under review: (1) the bias in conducting research, (2) the indirectness of the evidence, and (3) the insufficiency of information. First, the included studies suffered from at least one major methodological limitation, mainly confounding, that makes their findings potentially biased. Second, in the majority of the studies, the media intervention was part of a multi-component intervention or part of larger programs, making the evidence indirect. Consequently, it was not possible to isolate the effects of media as stand-alone interventions. This approach to intervention design may be due to the fact that using media as stand-alone interventions might not be sufficient when aiming to impact policy [42], given the multiple and complex factors that affect the policy-making process [13]. One additional limitation is that in the survey and interview studies, the outcomes were assessed based on perceptions of respondents on effectiveness of media interventions and not actual evaluation of effectiveness.

**Table 2:** Identified Research Gaps

Domain of evidence gaps	Gaps identified
Study design	• Lack of well-designed comparative studies, particularly on social media
Intervention	• Limited evidence on effects of media interventions independent of other interventions (i.e., published studies assessed media as part of multi-component intervention) • Limited evidence on planned media interventions (i.e., most published studies assessed media coverage that was not planned) • Limited description of media interventions assessed
Study setting	• Limited evidence from low and middle-income countries
Outcomes	• Lack of rigorous evaluation of outcomes (e.g., assessed based on perceptions of respondents about effectiveness of media interventions and not actual evaluation of effectiveness) • Limited evidence on the impact on policy stages • Lack of studies evaluating the policy evaluation stage

The aforementioned challenges and limitations of the included studies made it difficult to make any inferences on effectiveness as the authors of these papers do not explicitly provide an evidence for the link between the intervention and the outcome. Thus, we reported the results cautiously and relied on what the authors of the included papers reported. Third, our review showed limited quantity of research on the effects of planned media interventions and social media in particular on health policy-making.

Another major challenge in the reporting of most of the included studies is the very limited description of the media interventions, knowing that these qualify as complex interventions. This makes it difficult to understand the specific media intervention that was tested, and what component of that complex intervention might have been effective. Indeed, two other systematic reviews examining the effectiveness of mass media on health services utilization [10] and on reducing alcohol-impaired driving and alcohol-related crashes [53] identified similar limitations in terms of the description of the intervention.

There is also a dearth of studies reporting on the effectiveness of media interventions on health policies in low middle-income countries (LMIC). This may be linked to the fact that the production of Health Policy and Systems Research (HPSR) is still in its infancy in these countries [54–56].

We are aware of a number of systematic reviews assessing the use of media/social media in clinical medicine and public health. For instance, media interventions were assessed for their impact on smoking cessation [8], reducing the risk of alcohol-related injuries or crashes [53], increasing child survival in LMICs [57], and changing health behavior in fields such as HIV prevention [20, 58] and physical activity [19, 59]. Two reviews identified evidence that mass media campaigns can in fact have positive impact [8, 57]. Patel et al. showed that using social media, especially Facebook and blogs, likely improves care for patients with chronic disease [60]. Stellefson et al. reported that Web 2.0 can benefit older adults in managing their diseases [61]. Social media were also shown to be used for a variety of conditions and purposes in child health [62].

### Implications for research and policy

This systematic review can inform researchers and funders interested in understanding the interaction between the media and the policy worlds. Researchers are encouraged to conduct more and better-designed primary research studies on social media interventions given the rise of their use in the recent decade and potential impact on policy-making. Researchers should develop and follow guidelines for designing and evaluating complex media interventions. Moreover, there is a need for better reporting of studies in this field, taking into consideration guidelines for the reporting of complex interventions when describing the media intervention used [63–65].

This systematic review highlighted the challenges of evaluating the impact of media advocacy on health policy given the difficulties in applying experimental methods, the complex nature of these interventions, and the multiple factors influencing the policy-making process [13, 26]. Future primary studies and systematic reviews should include process evaluation and qualitative components to explore factors behind successes and failures, the impact of the context, and elements of best practices in health policy-making media campaigns [66]. Furthermore, future research should focus on the influence of media on health policy-making in diverse settings. Examining the role of media in these LMICs is important to design context-specific strategies and understand how the impact of media campaigns and interventions can differ across various health systems and socioeconomic realities. Funding agencies are also called to support future studies particularly in LMICs, where research production is still at its early stages, and support capacity building for research in the field of HPSR.

Our findings can also inform, to some extent, civil society organizations, advocacy groups, and researchers working to influence policy-making when considering media as tools for policy change. One particular important finding that emerged is the unintended effects of media campaigns to energize opponents. This implies that any media intervention has to be carefully designed and thought through to take into account such situations.

### Strengths and limitations

This review has two main strengths. First, and to our knowledge, this is the first systematic review examining the effects of planned media interventions, including social media, on health policy-making. Second, we have conducted the review using standard, explicit, and rigorous methods [67] and we followed standard methods for reporting systematic reviews [68]. This includes a very comprehensive search, including of the Communication and Mass Media Complete database, the main database for media-related articles. One limitation of this review is the use of stages heuristic framework while some academics consider it to assume linearity of the public policy process that does not exist in real world. However, it did help us synthesize the identified evidence by providing a simplified and useful way in viewing the entire public policy process [24].

## Additional files

Additional file 1: Search strategies for electronic databases. (DOCX 24 kb)

Additional file 2: Included case studies. (DOCX 35 kb)

Additional file 3: Methodological appraisal. (DOCX 23 kb)

Additional file 4: Results table. (DOCX 22 kb)
