# Supplementary material for: Using media to impact health policy-making: an integrative systematic review
Source: Implement Sci. 2017 Apr 18;12:52. doi: 10.1186/s13012-017-0581-0 (PMC5395744; doi:10.1186/s13012-017-0581-0)
Supplement: Supplementary file 1 — Search strategies for electronic databases. (DOCX 24 kb) [file 13012_2017_581_MOESM1_ESM.docx]

**Additional file 1:**

**Appendix 1: Search strategies for electronic databases**

**Medline search strategy:**

Database: Ovid MEDLINE(R) without Revisions <1996 to June Week 2 2015>

Search Strategy:

--------------------------------------------------------------------------------

1 Social Media/ (1671)

2 (social adj2 (medium or media or medias)).tw. (1458)

3 Social Networking/ (1039)

4 (social adj2 (networking or net-working)).tw. (962)

5 facebook.tw. (712)

6 (twitter or tweet*).tw. (542)

7 Blogging/ (597)

8 (blog* or microblog* or weblog*).tw. (745)

9 ("web 2.0" or "web 3.0").tw. (372)

10 (youtube or flickr or tumblr or linkedin or instagram or vine or (google adj plus)).tw. (1273)

11 communications media/ or exp mass media/ or radio/ or television/ or Newspapers/ (23199)

12 (media adj2 (print or printed or news or mass or coverage or communication* or broadcast* or broad-cast* or report or reports or reporting* or message* or campaign*)).tw. (5391)

13 (news adj2 (print or printed or coverage or report or reports or reporting)).tw. (630)

14 (press adj2 (release* or media or coverage or printed or print)).tw. (421)

15 (newspaper* or news-paper* or television* or televised or journalis*).tw. (9191)

16 ((radio or radios) adj2 (broadcast* or broad-cast* or news or station* or campaign* or listener* or communication* or message* or program*)).tw. (409)

17 or/1-16 (37255)

18 policy/ or smoke-free policy/ or social control policies/ or public policy/ or health policy/ or health care reform/ or lobbying/ or Jurisprudence/ or environmental policy/ or family planning policy/ or nutrition policy/ or legislation as topic/ or exp legislation, drug/ or legislation, food/ or legislation, hospital/ or legislation, medical/ or legislation, nursing/ or legislation, pharmacy/ or social control, formal/ or government regulation/ or law enforcement/ (122041)

19 child advocacy/ or consumer advocacy/ or patient advocacy/ (15732)

20 exp policy making/ (15530)

21 Decision Making/ (51606)

22 (decision* adj (making or maker*)).tw. (59753)

23 (clinical or treatment).tw. (2961612)

24 (21 or 22) not 23 (56218)

25 ((health* adj2 reform*) or lobby* or advoca*).tw. (41458)

26 (agenda* adj2 setting*).tw. (297)

27 ((government* or public) adj2 decision*).tw. (900)

28 (government* adj2 regulation*).tw. (641)

29 (law or laws or legal* or legislation* or decree* or jurisprudence).tw. (85292)

30 policymak*.tw. (4467)

31 (policy or policies).tw. (107775)

32 18 or 19 or 20 or 24 or 25 or 26 or 27 or 29 or 30 or 31 (359801)

33 17 and 32 (4483)

34 limit 33 to yr="2005 - 2015" (3072)

***************************

**Embase search strategy**

Database: Embase <1980 to 2015 Week 25>

Search Strategy:

--------------------------------------------------------------------------------

1 Social Media/ (4147)

2 (social adj2 (media or medias or medium)).ti,ab. (3079)

3 (social adj2 (network* or net-work*) adj2 (site* or website*)).ti,ab. (796)

4 (facebook or twitter or tweet*).ti,ab. (2373)

5 (blog* or microblog* or weblog*).ti,ab. (1484)

6 ("web 2.0" or "web 3.0").ti,ab. (552)

7 (youtube or flickr or tumblr or linkedin or instagram or vine or (google adj plus)).ti,ab. (2167)

8 exp mass medium/ or exp television/ (26864)

9 (media adj2 (print or printed or news or mass or coverage or communication* or broadcast* or broad-cast* or reporting* or message* or campaign*)).ti,ab. (8521)

10 (news adj2 (print or printed or coverage or reporting*)).ti,ab. (470)

11 (newspaper* or news-paper* or television* or televised or journalist*).ti,ab. (17561)

12 ((radio or radios) adj2 (broadcast* or broad-cast* or news or station* or campaign* or listener* or communication* or program*)).tw. (726)

13 or/1-12 (50299)

14 exp health care policy/ or exp smoking ban/ or exp law/ or exp law enforcement/ or jurisprudence/ or government regulation/ (272424)

15 consumer advocacy/ or child advocacy/ or patient advocacy/ (24907)

16 (policy or policies or policymak* law or laws or legal* or legislation* or decree* or jurisprudence).ti,ab. (297677)

17 ((health or healthcare) adj2 reform*).ti,ab. (14281)

18 (agenda* adj2 setting*).ti,ab. (475)

19 (lobby* or advoca*).ti,ab. (62216)

20 (decision* adj (making or maker*)).ti,ab. (104419)

21 ((government* or public) adj2 decision*).ti,ab. (1481)

22 (government adj2 regulation*).ti,ab. (975)

23 or/14-21 (644785)

24 13 and 23 (6520)

25 limit 24 to yr="2005 -Current" (4371)

***************************

**Communication and Mass Media Complete**

| **#** | **Query** | **Limiters/Expanders** |
| --- | --- | --- |
| S22 | S9 AND S15 AND S20 | Limiters - Published Date: 20050101-20151231  Search modes - Boolean/Phrase |
| S21 | S9 AND S15 AND S20 | Search modes - Boolean/Phrase |
| S20 | S16 OR S17 OR S18 OR S19 | Search modes - Boolean/Phrase |
| S19 | TI (physical N2 activit*) OR AB (physical N2 activit*) OR SU (physical N2 activit*) OR KW (physical N2 activit*) | Search modes - Boolean/Phrase |
| S18 | TI (road* N2 safety) OR AB (road* N2 safety) OR SU (road* N2 safety) OR KW (road* N2 safety) | Search modes - Boolean/Phrase |
| S17 | TI (Quality N2 (improvement* or care or standard* or control or assurance)) OR AB (Quality N2 (improvement* or care or standard* or control or assurance)) OR SU (Quality N2 (improvement* or care or standard* or control or assurance)) OR KW (Quality N2 (improvement* or care or standard* or control or assurance)) | Search modes - Boolean/Phrase |
| S16 | TI (Health* or pharmaceutical* or pharmacist* or pharmacy or pharmacies or drug* or medica* or medicines or tobacco or smoking or smoke or hookah or shisha or waterpipe* or alcohol* or nurs* or midwives or midwife or doctor* or physician* or dentist* or environment* or pollution* or nutrition* or food or obesit* or dietary or vaccin* or immuniz* or immunis* or breastfeeding or hospital* or patient* or HIV or HIV/AIDS or disease*) OR AB (Health* or pharmaceutical* or pharmacist* or pharmacy or pharmacies or drug* or medica* or medicines or tobacco or smoking or smoke or hookah or shisha or waterpipe* or alcohol* or nurs* or midwives or midwife or doctor* or physician* or dentist* or environment* or pollution* or nutrition* or food or obesit* or dietary or vaccin* or immuniz* or immunis* or breastfeeding or hospital* or patient* or HIV or HIV/AIDS or disease*) OR SU (Health* or pharmaceutical* or pharmacist* or pharmacy or pharmacies or drug* or medica* or medicines or tobacco or smoking or smoke or hookah or shisha or waterpipe* or alcohol* or nurs* or midwives or midwife or doctor* or physician* or dentist* or environment* or pollution* or nutrition* or food or obesit* or dietary or vaccin* or immuniz* or immunis* or breastfeeding or hospital* or patient* or HIV or HIV/AIDS or disease*) OR KW (Health* or pharmaceutical* or pharmacist* or pharmacy or pharmacies or drug* or medica* or medicines or tobacco or smoking or smoke or hookah or shisha or waterpipe* or alcohol* or nurs* or midwives or midwife or doctor* or physician* or dentist* or environment* or pollution* or nutrition* or food or obesit* or dietary or vaccin* or immuniz* or immunis* or breastfeeding or hospital* or patient* or HIV or HIV/AIDS or disease*) | Search modes - Boolean/Phrase |
| S15 | S10 OR S11 OR S12 OR S13 OR S14 | Search modes - Boolean/Phrase |
| S14 | TI (Decision N1 (maker* or making)) OR AB (Decision N1 (maker* or making)) OR SU (Decision N1 (maker* or making)) OR KW (Decision N1 (maker* or making)) | Search modes - Boolean/Phrase |
| S13 | TI (Public N2 decision*) OR AB (Public N2 decision*) OR SU (Public N2 decision*) OR KW (Public N2 decision*) | Search modes - Boolean/Phrase |
| S12 | TI (Government* N2 (decision* or regulation*)) OR AB (Government* N2 (decision* or regulation*)) OR SU (Government* N2 (decision* or regulation*)) OR KW (Government* N2 (decision* or regulation*)) | Search modes - Boolean/Phrase |
| S11 | TI (Agenda* N2 setting*) OR AB (Agenda* N2 setting*) OR SU (Agenda* N2 setting*) OR KW (Agenda* N2 setting*) | Search modes - Boolean/Phrase |
| S10 | TI (Policy or policies or policymak* or law or laws or legal* or legislation* or decree* or jurisprudence or lobby* or advoca*or reform*) OR AB (Policy or policies or policymak* or law or laws or legal* or legislation* or decree* or jurisprudence or lobby* or advoca*or reform*) OR SU (Policy or policies or policymak* or law or laws or legal* or legislation* or decree* or jurisprudence or lobby* or advoca*or reform*) OR KW (Policy or policies or policymak* or law or laws or legal* or legislation* or decree* or jurisprudence or lobby* or advoca*or reform*) | Search modes - Boolean/Phrase |
| S9 | S1 OR S2 OR S3 OR S4 OR S5 OR S6 OR S7 OR S8 | Search modes - Boolean/Phrase |
| S8 | TI (press N2 (release* or media or coverage or printed or print)) OR AB (press N2 (release* or media or coverage or printed or print)) OR SU (press N2 (release* or media or coverage or printed or print)) OR KW (press N2 (release* or media or coverage or printed or print)) | Search modes - Boolean/Phrase |
| S7 | TI ((radio or radios) N2 (broadcast* or broad-cast* or news or station* or campaign* or listener* or communication* or program*)) OR AB ((radio or radios) N2 (broadcast* or broad-cast* or news or station* or campaign* or listener* or communication* or program*)) OR SU ((radio or radios) N2 (broadcast* or broad-cast* or news or station* or campaign* or listener* or communication* or program*)) OR KW ((radio or radios) N2 (broadcast* or broad-cast* or news or station* or campaign* or listener* or communication* or program*)) | Search modes - Boolean/Phrase |
| S6 | TI (newspaper* or news-paper* or television* or televised or journalis*) OR AB (newspaper* or news-paper* or television* or televised or journalis*) OR SU (newspaper* or news-paper* or television* or televised or journalis*) OR KW (newspaper* or news-paper* or television* or televised or journalis*) | Search modes - Boolean/Phrase |
| S5 | TI (news N2 (print or printed or coverage or reporting*)) OR AB (news N2 (print or printed or coverage or reporting*)) OR SU (news N2 (print or printed or coverage or reporting*)) OR KW (news N2 (print or printed or coverage or reporting*)) | Search modes - Boolean/Phrase |
| S4 | TI (media N2 (mass or print or printed or news or coverage or communication* or broadcast* or broad-cast* or reporting* or message* or campaign*)) OR AB (media N2 (mass or print or printed or news or coverage or communication* or broadcast* or broad-cast* or reporting* or message* or campaign*)) OR SU (media N2 (mass or print or printed or news or coverage or communication* or broadcast* or broad-cast* or reporting* or message* or campaign*)) OR KW (media N2 (mass or print or printed or news or coverage or communication* or broadcast* or broad-cast* or reporting* or message* or campaign*)) | Search modes - Boolean/Phrase |
| S3 | TI (facebook* or twitter or tweet* or blog* or microblog* or weblog* youtube or flickr or tumblr or linkedin or Instagram or vine or “google plus” or “web 2.0” or “web 3.0”) OR AB (facebook* or twitter or tweet* or blog* or microblog* or weblog* youtube or flickr or tumblr or linkedin or Instagram or vine or “google plus” or “web 2.0” or “web 3.0”) OR SU (facebook* or twitter or tweet* or blog* or microblog* or weblog* youtube or flickr or tumblr or linkedin or Instagram or vine or “google plus” or “web 2.0” or “web 3.0”) OR KW (facebook* or twitter or tweet* or blog* or microblog* or weblog* youtube or flickr or tumblr or linkedin or Instagram or vine or “google plus” or “web 2.0” or “web 3.0”) | Search modes - Boolean/Phrase |
| S2 | TI (social N2 (networking or net-working)) OR AB (social N2 (networking or net-working)) OR SU (social N2 (networking or net-working)) OR KW (social N2 (networking or net-working)) | Search modes - Boolean/Phrase |
| S1 | TI (social N2 (media or medium or medias)) OR AB (social N2 (media or medium or medias)) OR SU (social N2 (media or medium or medias)) OR KW (social N2 (media or medium or medias)) | Search modes - Boolean/Phrase |

**WHO Global Health Library:**

(Facebook* or twitter or tweet* or blog or blogs or blogging or microblog* or micro-blog* or weblog* or youtube or flickr or tumblr or linkedin or Instagram or (web 2.0) or (google plus) or (social media) or (social medias) or (social networking website*) or (social networking site*) or (online social networking) or (social net-working website*) or (social net-working site*) or (online social net-working) or (mass media) or (print media) or (printed media) or (news media) or (media coverage*) or (media communication*) or (broadcast* media) or (broad-cast* media) or (media reporting*) or (media message*) or (media campaign*) or (print news) or (printed news) or (news coverage) or (news reporting) or jounalis*or newspaper* or news-paper* or television* or televised or (radio broadcast*) or (radio broad-cast*) or (radio station*) or (radio campaign*) or (radio program*) or (radio communication*)) and (policy or policies or policymak*or law or laws or legislation* or legal* or decree* or jurisprudence or (agenda* setting) or advocacy or advocacies or advocate or advocating or lobby* or (health reform*) or (government regulation*) or (government decision*) or (public decision*))

**Cochrane Central Register of Controlled Trials (CENTRAL)**

(Facebook* or twitter or tweet* or blog* or microblog* or micro-blog* or weblog* or youtube or flickr or tumblr or linkedin or vine or Instagram or (web 2.0) or (google plus) or (social media) or (social medias) or (social networking website*) or (social networking site*) or (online social networking) or (social net-working website*) or (social net-working site*) or (online social net-working) or (mass media) or (print media) or (printed media) or (news media) or (media coverage*) or (media communication*) or (broadcast* media) or (broad-cast* media) or (media reporting*) or (media message*) or (media campaign*) or (print news) or (printed news) or (news coverage) or (news reporting) or jounalis*or newspaper* or news-paper* or television* or televised or (radio broadcast*) or (radio broad-cast*) or (radio station*) or (radio campaign*) or (radio program*) or (radio communication*)) and (policy or policies or policymak*or law or laws or legislation* or legal* or decree* or jurisprudence or (agenda* setting) or advocacy or advocacies or advocate or advocating or lobby* or (health reform*) or (government regulation*) or (government decision*) or (public decision*) or (decision* making) or (decision* maker*))
