# Supplementary material for: Using media to impact health policy-making: an integrative systematic review
Source: Implement Sci. 2017 Apr 18;12:52. doi: 10.1186/s13012-017-0581-0 (PMC5395744; doi:10.1186/s13012-017-0581-0)
Supplement: Supplementary file 2 — Included case studies. (DOCX 35 kb) [file 13012_2017_581_MOESM2_ESM.docx]

**Additional file 2, Appendix 2: Included Case Studies**

Summary of included case studies:

Six case studies were from high income countries (United States (n= 3), UK (n=2) and Australia (n=1)) while 5 were from low and middle income countries (LMICs) (India (n=2), Mexico (n=1), Nigeria (n=1) and Republic of Moldova (n=1)). The health topics were oral health (n=1), alcohol control (n=1), tobacco control (n=4), road safety (n=1), maternal and child health (n=1), childhood Asthma (n=1), Gestational diabetes mellitus (n=1) and physical activity (n=1). The media intervention was part of multi-component campaigns, in all but three cases where it was implemented alone [[34](#_ENREF_34) [36](#_ENREF_36) [38](#_ENREF_38)].Two campaigns relied on the use of social media [[33](#_ENREF_33) [38](#_ENREF_38)], while the remaining ones used traditional media. The case studies examined agenda-setting (n=3), policy adoption (n=9) and implementation (n=6). None looked at policy formulation or evaluation.

| **Study name and country** | **Health topic** | **Type of media** | **Media strategies/intervention** | **Policy outcome** |
| --- | --- | --- | --- | --- |
| Arnott 2007  United Kingdom | Tobacco control | Print and broadcast media (TV, radio and press coverage) | Advocacy campaign including positive media coverage:  *“The media plan included utilizing a range of media opportunities, opinion polls and surveys, and profile raising events.* *Central to the effective media strategy was finding ways of promoting the evidence base for the harm caused by secondhand smoke.* *Tactics also involved using medical and scientific experts expressing their concerns at profile raising events, and the exploitation of reactive opportunities such as publications of international research into secondhand smoke.”* | **Policy adoption**  Adoption of smoke-free legislation:  *“On 14 February 2006, the House of Commons voted by a majority of 200 for comprehensive smoke-free legislation. Subsequently the smoke-free legislation came into force very successfully on 1 July 2007.”* |
| Bhojani 2013  India | Tobacco control | Print and broadcast media (television and radio) | Advocacy campaign that included:  An administrative and political advocacy, media advocacy, legal advocacy and community mobilization. The objectives of the campaign were: “*(1) to demand withdrawal of government’s sponsorship of and participation in Global Tobacco Networking Forum (GTNF) organized by the USA-based tobacco industry magazine; (2) to ensure that GTNF proceedings comply with Cigarette and Other Tobacco Products act and (3) to prevent any such future sponsorship by government.”*  Media advocacy consisted of earned media coverage through constant interactions with journalists, dissemination of press releases and personal communication with journalists. | **Policy adoption**  Policy withdrawal:    *“Government withdrew participation and financial sponsorship from the tobacco industry event.”* |
| Gomm 2006  Australia | Road safety | Print media | Campaign including media advocacy:  Media was used to promote a road safety campaign (the Chips on Rail campaign) that aims to influence public opinion and also opinion leaders and policy-makers.  Media advocacy consisted of “*gaining media access that provided visibility, credibility and legitimacy to the campaign and disseminating press releases announcing each meeting and activity, with follow-up after meetings to provide a strong communication link with the local media”.* | **Agenda-setting**  Raising the issue with policymakers  *“A local Member of Parliament and the Minister of Transport initiated meetings with the Alliance confirming that Albany Region Community Development Alliance (ARCDA) had been successful in raising the issue with key decision-makers.”*  **Policy adoption and implementation**  Establishing a rail link to reduce traffic and improve road safety  *“The goal of providing a rail link was achieved and continues to be used by one timber company”.* |
| Jernigan 2011  United States | Alcohol control | Print, broadcast and electronic media (television, radio, magazines, and the Internet) | Advocacy including media advocacy:  Media was used to disseminate research findings. “*By using media advocacy to disseminate its findings, the Center kept*  *a very public focus on the industry's practices, supporting advocacy efforts at national, state, and local levels to reduce youth exposure to alcohol advertising. Center staff and affiliates reached out to policymakers, using the research findings as a 'calling card'. Having its own databases permitted the Center to pinpoint its findings, producing market-specific analyses for television and radio advertising.”* | **Policy adoption and implementation**  Adopting and implementing alcohol control policies (e.g. underage drinking and alcohol advertising issues)  *“generated legislative resolutions”*  *“Banned alcohol advertising on bus shelters (Philadelphia)”*  *“placed industry's voluntary restriction on proximity of billboards advertising alcohol*  *to schools, churches, and playgrounds into state administrative rules so that state officials could enforce them”*  *“worked to re-classify alcopops from beer to distilled spirits to make them more expensive and less accessible (California, Utah, Nebraska, Maine)”*  *“used a youth-generated Alcohol Retailer's Local Marketing Code of Conduct to call attention to good and bad practices in point-of-purchase marketing (Maine)”* |
| Kreger 2011  United States | Childhood Asthma | Not clear | Advocacy including media advocacy  Media and data were used to*” educate, raise awareness, and promote policy change in their communities. Coalitions developed multistep media strategies to introduce and frame issues, define necessary next steps, and obtain support from stakeholders.”* | **Policy adoption and implementation**  Adoption and implementation of Environmental Justice Policies  *“Large policy changes included state or local level legislation or changes in regulations”* |
| Lane 2012  Mexico | Tobacco control | Print media  Broadcast media  Social media  Electronic media | Campaign including media advocacy. Media advocacy including media pressure and paid and earned media:   - Media pressure was applied specifically to different areas of the government as the draft legislation moved from the Chamber of Deputies to the Senate, and then to the President’s desk for final approval. - Earned media: Press releases, political and academic forums, public, demonstrations, report releases, letters to public officials that were also distributed to media outlets, letters to the editors of key newspapers, and radio and television interviews - Paid media: Billboards, banner ads and murals; blog outreach; online media via social networks such as Facebook and Twitter; and a website. | **Policy adoption and implementation**  Adoption of policy for increasing taxes on tobacco products  *“In one year, votes (chamber of deputies, senate) went from 191 in favor to 500 in favor.”*  *“The 2010 tax increase was a major victory for tobacco control, particularly when viewed in light of the previous year’s resounding defeat.”*  *“According to economic simulation models, the tax increase is projected to reduce tobacco consumption by 17.3%, an overwhelming country-wide health impact.”* |
| Madhab 2011  India | Gestational diabetes mellitus | Print, broadcast and electronic media (radio, mobile SMS) | A multimedia awareness and advocacy campaign  The campaign aims at mainstreaming gestational diabetes mellitus (GDM) in the health delivery system.  *“Key messages are disseminated through a multimedia approach including print, radio, wall paintings, mobile SMS, internet, and a van with IEC materials and trained community mobilisers.”* | **Policy implementation**  Implementing policies on Gestational diabetes mellitus  *“Screening for GDM has been added to the list of free services to be provided to women below the poverty line on the maternal and child healthcare card used in the NRHM Program.”*  *“The state governments of Delhi and Punjab announced that screening for diabetes in pregnant women would become mandatory in their respective states”*  *“The government of Bihar has made GDM screening mandatory in its public health facilities and should be seen as a multiplier effect.”* |
| Okonofua 2011  Nigeria | Maternal and child health | Broadcast media (television) and detailed media publicity (type not clear) | Advocacy consisted of:  *“Public presentation on Maternal and child health to high-level policymakers, dissemination of situational analysis report, and media publicity”. “The involvement of the media that gave wide publicity to the related advocacy activities.”* | **Agenda setting**  Building the commitment of high-level government officials in addressing maternal and child health  *“The ruling Peoples Democratic Party included the policy in its 2007 electioneering campaign manifesto and directed its State Governors to implement the policy.*  *This preliminary report showed that advocacy has been successful in building the commitment of high-level government officials in addressing maternal and child health in Nigeria.”*  **Policy adoption and implementation**  Adoption of a policy of free treatment for pregnant women and children at all tertiary health institutions  *“Upon presentation of the needs assessment report to the Federal*  *Executive Council, the President immediately declared a policy of free treatment for pregnant women and children at all tertiary health institutions in Nigeria. However, the policy is yet to be implemented due to transition to a new administration at the Federal level in May 2007. Thus, since the advocacy began, more states began implementing free maternal and child health policies.*  *“Not only has the federal government declared a policy of free treatment for mothers and children, additional seven States started implementing comprehensive free maternal and child health program within 6 months of the advocacy activities.”* |
| Tataru 2009  Republic of Moldova | Tobacco control | Print media  Broadcast media  Electronic media | Media advocacy campaign that aims at:   1. Involving mass media and improving the knowledge of journalists 2. Raising public and decision makers awareness (through the media) to promote public policy initiatives and obtaining a change social. | **Policy adoption**  Approval of tobacco control law  *“Thus, media advocacy has contributed significantly to the*  *to the unfolding of political and social events in this field (approval of FCTC; approval of a new Law on tobacco and tobacco products, “stipulating ,restrictions and afferent*  *provisions to the consumption of tobacco products and the harmful effects on health””* |
| Vaughan 2009  United States | Oral Health | Print media  (Newspapers, letters to editors)  Paid media activity | Campaign including the use paid and earned media:  *“The purpose of the Watch Your Mouth campaign is to help advocates and experts reframe children’s oral health in order to advance public policy solutions to this widespread problem. The campaign builds on the power of community and policy advocacy by coordinating these strategies with extensive opinion pieces in newspapers and letters to the editor, along with paid media activity.”* | **Policy Adoption**  Passing of oral health bill  *“In addition to shifts in public understanding and support, Massachusetts made important policy advances over this period, from reinstating Medicaid Dental benefits for all adults in the state, to passing an omnibus oral health bill that mandated a state office of oral health with a state dental director, and created new classifications of oral health professionals that will help to increase access to care.”* |
| Weiler 2013  United Kingdom | Physical activity | Social media  (blogs, twitter) | Use of Social media:  *“The plan was to use social media and electronic mail to try and change the draft priorities by seeking as much feedback as possible to the open consultation in support of including a physical activity priority. Our goal was to inform and interest as many people as possible about the perceived problem by using ‘viral marketing’ techniques in the hope that they would be guided to leave feedback to the open consultation.”* | **Agenda-setting**  “Increasing physical activity and promoting a healthy weight” as a priority in Health and Wellbeing Strategy for 2013-16  *“Hertfordshire’s new Health and Wellbeing Strategy for 2013-16 was launched on Monday 11 February, 2013 and now includes the following priority in the revised strategy “Increasing physical activity and promoting a healthy weight.”* |
