# Supplementary material for: Using media to impact health policy-making: an integrative systematic review
Source: Implement Sci. 2017 Apr 18;12:52. doi: 10.1186/s13012-017-0581-0 (PMC5395744; doi:10.1186/s13012-017-0581-0)
Supplement: Supplementary file 3 — Methodological appraisal. (DOCX 23 kb) [file 13012_2017_581_MOESM3_ESM.docx]

**Additional file 3**

**Appendix 3: Methodological appraisal**

1. Assessment of methodological quality of qualitative studies using the CASP tool (n=1)

| Study Title | Year | Was there a clear statement of the aims of the research? | Is the qualitative methodology appropriate? | Was the research design appropriate to address the aims of the research? | Was the recruitment strategy appropriate to the aims of the research? | Was the data collected in a way that addressed the research issue? | Has the relationship between researcher and participants been adequately considered? | Have ethical issues been taken into consideration? | Was the data analysis sufficiently rigorous? | Is there a clear statement of findings? | How valuable is the research? |
| --- | --- | --- | --- | --- | --- | --- | --- | --- | --- | --- | --- |
| Haq | 2010 | yes | yes | yes | yes | yes | No | Can’t tell | yes | yes | yes |

1. Methodological quality of quantitative studies using surveys (n=3) (tool adapted from Lotfi et al.)

| **Study ID** | **Sample size calculation** | **Sampling:**   - **Frame:** - **Method (type)** | **Recruitment method** | **Response rate** | **Administration method** | **Validity of tool**  **Pilot testing done** |
| --- | --- | --- | --- | --- | --- | --- |
| Gardner 2010 | Not reported | Not reported | Not reported | Not reported | Not reported | - Validity of tool: Not reported - Pilot-testing: Not reported |
| Gowda 2008 | Not reported | Frame: telephone directory  Method: random sampling | Telephone surveys | reported | Telephone surveys | - Validity of tool: Not reported - Pilot-testing: Not reported |
| Sivaneswaran 2011 | Reported | Frame:  A random sample of 944 out of 7582 households was selected from a telephone directory (Desktop Marketing Systems Pty Ltd: Marketing Pro)  Method: random sampling | Not reported | reported | - Telephone interviews - face-to-face interview | - Validity of tool: Not reported - Pilot-testing: Not reported |

1. Risk of bias of controlled before and after study, using the EPOC risk of bias criteria for the controlled before and after studies (n=1)

| Study name | Was the allocation sequence adequately generated? | Was the allocation adequately concealed? | Were baseline outcome measurements similar? | Were baseline characteristics similar? | Were incomplete outcome data adequately addressed? | Was knowledge of the allocated interventions adequately prevented during the study? | Was the study adequately protected against contamination? | Was the study free from selective outcome reporting? | Was the study free from other risks of bias? |
| --- | --- | --- | --- | --- | --- | --- | --- | --- | --- |
| Rehnman 2005 (purchase studies) | High risk | High risk | Unclear risk | Low risk | Unclear risk | Unclear risk | High risk | Low risk | High risk |

1. Limitations of the remaining 5 studies*:

| **Study name** | **Study design** | **Limitations** |
| --- | --- | --- |
| Harwood 2005 | Media and document analysis | The study did not adjust for potential confounding |
| Leurer 2009 | Media analysis | The effectiveness of the media intervention was only tested through an analysis of the print media coverage of events and the press releases |
| Niederdeppe 2007 | Event history analysis | The authors could not control for (or estimate) the effect of formal presentations of several SWAT groups to county officials to gain support for TPPOs. In addition, authors reported that the county-level participation measures may not have captured all the effects of community mobilization. |
| Rock 2011 | Content analysis | The study assessed the effectiveness of the media intervention qualitatively. Another limitation is the post hoc nature of the data collection. |
| Vasudevan et al. 2009 | Uncontrolled before and after | The study assessed the effectiveness of media as part of multi-component intervention (coupled with enforcement campaign) and not independently. The study did not adjust for confounding |

* We were not able to find tools to assess the methodological quality of the five studies included in this table; we reported the limitations narratively in this table.
