# Supplementary material for: Using media to impact health policy-making: an integrative systematic review
Source: Implement Sci. 2017 Apr 18;12:52. doi: 10.1186/s13012-017-0581-0 (PMC5395744; doi:10.1186/s13012-017-0581-0)
Supplement: Supplementary file 4 — Results table. (DOCX 22 kb) [file 13012_2017_581_MOESM4_ESM.docx]

**Additional file 4, Appendix 4: Results table**

| Study ID | Policy Outcome type, measurement and theme | Results |
| --- | --- | --- |
| Rehnman 2005 | Policy implementation: assessed as successful beer purchase attempts  Statistical evaluation  *“Media as awareness tools to improve compliance with laws and regulations ”* | **Purchase study:**  *“In the baseline study, 66% of the purchase attempts in the intervention area and 60% in the comparison area led to purchases. In the first follow-up study a year later, 73% of all purchase attempts in the intervention area led to purchases. The increase in the intervention area from 66% to 73% was not statistically significant, whereas the increase in the comparison area from 60% to 86% was significant; d526.2 (95% confidence intervals 12.8–39.5). Purchases were significantly more common in the comparison area compared with the intervention area, 86% versus 73%; d513.3 (95% CI 0.5–26.1). At the second follow-up study 2 years later, a significant decrease in availability was noted in both the intervention area and in the comparison area. In the intervention area, the decrease in purchases was 29% and in the comparison area 42%. There were no significant differences in reduction between the two areas. None of the shops that sold beer at the first purchase attempt and then were visited by a parent sold beer at the second attempt.”* |
| Vasudevan 2009 | Policy implementation: assessed as change in seat belt usage rate  Statistical evaluation  *“Media as awareness tools to improve compliance with laws and regulations ”* | *“Table VI shows that the overall seat belt usage rate in 2005 in the state of Nevada increased from 88.4 percent before the media and enforcement campaigns to 94.8 percent after the campaigns. The seat belt usage rate for drivers increased from 87.7 to 94.4 percent, whereas for front seat passengers alone the increase was from 90.1 to 96.0 percent. The seat belt usage rates in 2005 for drivers based on gender showed that the usage rate for male drivers increased from 86.0 to 93.6 percent and for female drivers the increase was from 91.3 to 96.2 percent. All of these are highly significant statistically. Therefore, in all cases the null hypothesis is rejected, indicating that the seat belt rates after the campaigns are statistically greater than the seat belt rates before the campaigns. Analyses of the data for the years 2004 and 2003 also show similar increases in seat belt usage rates.”*  *“The results reported herein show that by effectively coupling media and enforcement campaigns, a significant increase in seat belt usage could be achieved in Nevada, a state with a secondary seat belt law.”* |
| Sivaneswaran 2011 | Policy adoption:  assessed as adoption of water fluoridation policy  Quantitative description  *“Media as awareness tools to gain public support leading to policy adoption”* | *“The most common sources where respondents had gained information regarding water fluoridation were the newspaper (n=235), followed by the radio (n=115), word of mouth (n=85) and television (n=80). In addition, the survey indicated that 59% of participants who reported that they had gained information on fluoridation were supportive of the measure, compared with only 47% if they were not informed.”*  *“The role of the media as a source of information on water fluoridation cannot be underestimated in any fluoridation campaign. This was confirmed by the WRI survey which reported that the newspaper and radio were the main sources of information from which respondents learnt about fluoridation.”*  *In June 2005, the Mid-Western Regional Council resolved to implement water fluoridation in Mudgee and Gulgong.”*  *“Our experience in Mudgee and Gulgong demonstrate that with the use of a comprehensive, multifaceted approach in educating and consulting communities and stakeholders, it is possible to garner community support for water fluoridation and achieve a successful outcome in small rural communities”*  *“In Mudgee and Gulgong, the WRI survey found that if people were informed about water fluoridation, they were more likely to be supportive of the measure.”* |
| Leurer 2013 | Policy formulation:  assessed as change in government policy  Qualitative description  *“Media as advocacy tools to influence policy formulation”* | *“It is clear that the media advocacy efforts of nursing stakeholders in Saskatchewan in the 3-month period beginning January 21, 2000 increased public pressure for the government to reconsider its intended policy of imposing a 3-year diploma entry requirement despite opposition from the licensing body”.* |
| Haq 2010 | Agenda-setting:  assessed as change in policy behavior/approach  Qualitative description  *“Media as accountability tools leading to prioritizing and initiating policy discussions on Maternal and newborn health”* | *“Regarding the progress made on MNH issues in the districts since the airing of the programmes, half of the respondents reported having policy discussions on how to improve emergency care for mothers and newborns, and to raise funds for ensuring this care. Three fourths of the participants mentioned initiating analysis of the MNH situation in their districts.”*  *“Some 13 participants reported that they felt more accountable after having given on-camera commitments to improve MNH in their district. However, three Nazims felt that their TV appearance had little effect on their accountability status. Some 17 of the 20 respondents indicated that their resolve to tackle MNH issues had increased as a result of participating in this show. They further mentioned that MNH was a priority health issue for them as district policy makers and managers. In response to the question on participants' suggestions on improving such interventions for the future, some 14 out of the 20 participants indicated that this was a very effective strategy”*  *“The talk show format which was an innovative venture not tried previously in Pakistan and to the best of our knowledge nowhere in the developing world, appears to have been effective in influencing district health policy makers and setting MNH as a priority agenda in the respective districts”.* |
| Harwood 2005 | Policy adoption:  assessed as passage of alcohol bills  Quantitative description  *“Media as awareness tools leading to policy adoption ”* | *“Overall, our findings show that high press coverage was associated with unsuccessful alcohol bills; all successful bills under study, with the possible exception of a zero tolerance law, were enacted with little or no media attention. These findings suggest that press inattention has potential benefits for policy advocacy in at least two ways—to prevent mobilization of opponents and to permit stakeholders the opportunity to compromise during negotiations on bill content and wording”.* |
| Gowda 2008 | Policy adoption:  assessed as adoption of a new water fluoridation policy  Narrative description  *“Media as awareness tools to gain public support leading to policy adoption”* | *”Media advocacy involved both ”top down” and “bottom up” approaches, complementing adoption of the fluoridation position statement by the District Health Boards (DHB) and acting as a vehicle for reflecting community views on fluoridation.”*  *“The media played an important role during and after adoption of the position statement by DHB”*  *“The outcome of the telephone survey carried out in 2006 in Kaitaia and Kaikohe reflect the implications and effectiveness of fluoridation advocacy. It obtained a simple majority in favour of fluoridation and led directly to Far North District Council’s resolution to fluoridate Kaitaia and Kaikohe. Media advocacy is yet another key tool that played an important role in disseminating appropriate information. Analysis of articles and letters showed that media played an important role in linking all levels of the community including both proponents and opponents of water fluoridation and also spreading an effective message.”* |
| Niederdeppe 2007 | Policy adoption:  assessed as passage of tobacco product placement ordinances  Statistical evaluation  *“ Media as awareness tools leading to policy adoption”* | *“In support of H2, a one-unit increase in SWAT news exposure was associated with a 94% increase in the odds of counties enacting policy change (table 1, model 1). Other FTCP news exposure was not associated with changes in the odds of policy change. In addition, each quarter (starting from quarter 2, 1998) increased the odds of policy change by 33%.*  *None of the additional control variables included in model 2 was significant. The effect of previous-quarter SWAT news coverage remained significant and somewhat stronger in magnitude. A one-unit increase in SWAT news exposure was associated with a 134% increase in the odds of counties enacting an ordinance.”*  *“Higher levels of SWAT news coverage in the previous quarter increased the likelihood of a county enacting a TPPO. The effect persisted when controls for community mobilization and pro-tobacco marketing influences were included in the model.”* |
| Gardner 2010 | Policy adoption:  assessed as achieving a policy change or increased funding to clinics  Quantitative description  *“Media as awareness tools leading to policy adoption”* | *“Some grantees felt that the media activities did increase awareness of community health center issues by the public, funders, and policymakers.”*  *“Nearly all grantees (95%) consistently rated media advocacy as effective in increasing policymaker awareness. Only approximately 20%, however, reported that it had achieved a policy change or increased funding to clinics.”* |
| Rock 2011 | Agenda-setting:  Assessed as reactions of policymakers to media coverage  Qualitative description  “*Media as tools to increase policymakers’ awareness”* | *“Meanwhile, through e-mail, telephone and face-to-face communication, we learned that the media coverage had also generated interest within the Government of Canada, the Government of Alberta and among local providers of food relief and other social services.”*  *“The media strategy also sparked an inquiry from the Senate Committee on Social Affairs Subcommittee on Cities (documented in*  *an e-mail sent to M.R.), an inquiry from a political staff member (documented in an e-mail sent to K.T. and forwarded to M.R.) and discussion in the Alberta Legislature (documented in ‘Hansard’).”*  *“The questions posed by a Liberal opposition politician to Alberta’s Minister of Health reflected our key message i.e. ‘Poverty is a public health problem’. The reply from Alberta’s Minister of Health*  *(a member of the Legislative Assembly from the Progressive Conservative Party of Alberta) to this question was off-message, suggesting that ‘Food security is a personal responsibility’. In ‘a province that has opportunity for everyone’, he said, ‘the government is not responsible for what each one of 3.2 million Albertans eats’”* |
